# Technical note: how to determine the FDG activity for tumour PET imaging that satisfies European guidelines

**DOI:** 10.1186/s40658-016-0158-z

**Published:** 2016-09-29

**Authors:** Daniëlle Koopman, Jochen A. C. van Osch, Pieter L. Jager, Carlijn J. A. Tenbergen, Siert Knollema, Cornelis H. Slump, Jorn A. van Dalen

**Affiliations:** 1 Department of Nuclear Medicine, Isala, Zwolle, The Netherlands; 2 MIRA Institute for Biomedical Technology and Technical Medicine, University of Twente, Enschede, The Netherlands; 3 Department of Medical Physics, Isala, Zwolle, The Netherlands

**Keywords:** FDG-PET, Scan time protocol, Tumour imaging, EANM guidelines

## Abstract

**Background:**

For tumour imaging with PET, the literature proposes to administer a patient-specific FDG activity that depends quadratically on a patient’s body weight. However, a practical approach on how to implement such a protocol in clinical practice is currently lacking. We aimed to provide a practical method to determine a FDG activity formula for whole-body PET examinations that satisfies both the EANM guidelines and this quadratic relation.

**Results:**

We have developed a methodology that results in a formula describing the patient-specific FDG activity to administer. A PET study using the NEMA NU-2001 image quality phantom forms the basis of our method. This phantom needs to be filled with 2.0 and 20.0 kBq FDG/mL in the background and spheres, respectively. After a PET acquisition of 10 min, a reconstruction has to be performed that results in sphere recovery coefficients (RCs) that are within the specifications as defined by the EANM Research Ltd (EARL). By performing reconstructions based on shorter scan durations, the minimal scan time per bed position (*T*_min_) needs to be extracted using an image coefficient of variation (COV) of 15 %. At *T*_min_, the RCs should be within EARL specifications as well. Finally, the FDG activity (in MBq) to administer can be described by $A=c \cdot {w}^2\cdot \frac{T_{\min }}{t}$ with *c* a constant that is typically 0.0533 (MBq/kg^2^), *w* the patient’s body weight (in kg), and *t* the scan time per bed position that is chosen in a clinical setting (in seconds). We successfully demonstrated this methodology using a state-of-the-art PET/CT scanner.

**Conclusions:**

We provide a practical method that results in a formula describing the FDG activity to administer to individual patients for whole-body PET examinations, taking into account both the EANM guidelines and a quadratic relation between FDG activity and patient’s body weight. This formula is generally applicable to any PET system, using a specified image reconstruction and scan time per bed position.

## Background

Positron emission tomography/computed tomography (PET/CT) scanning, using the radioactive tracer fluor-18 fluordeoxyglucose (FDG), has an important role in tumour imaging for patients with cancer. There is a trend towards standardization and harmonization in FDG-PET scanning to allow comparisons of FDG uptake parameters across patients, scanners and medical centres [1]. Recently, version 2.0 of the European Association of Nuclear Medicine (EANM) procedure guidelines for FDG-PET tumour imaging was published. This guideline contains recommendations for tumour imaging with PET/CT by prescribing FDG activity as a function of a patient’s body weight, type of scanner, reconstruction method and scan duration [2].

It is widely known that PET image quality is influenced by a patient’s body weight. Heavier patients show more photon attenuation and higher scatter fractions, resulting in lower PET image quality for these patients when using a fixed tracer activity and scan time. This effect can be compensated by increasing the scan time and/or tracer activity in heavier patients [3–6]. De Groot et al. [7] demonstrated that the use of a dedicated FDG activity protocol, depending quadratically on a patient’s body weight, delivers a constant image quality across patients in several weight categories. Thereby, it provided an improved radiation exposure justification. This protocol has been included as an alternative in version 2.0 of the EANM procedure guidelines [2].

However, a practical approach on how to implement such a protocol in clinical practice is currently lacking. First, it is not clear how to translate minimum requirements for image quality into a quadratic formula that describes a patient-specific FDG activity for a given scanner, reconstruction method and scan duration. Second, when using a particular patient-specific FDG activity, it needs to be verified that the applied PET reconstruction meets the harmonizing specifications for recovery coefficients (RCs), as described on the EANM Research Ltd (EARL) website [8].

Our aim was to provide an easy applicable method that results in a formula describing the FDG activity to administer to a patient, that is quadratically related to a patient’s body weight and satisfies EANM procedure guidelines [2]. We intended to obtain a formula that is applicable to any PET system, using a specified image reconstruction and scan time per bed position.

## Methods

The formula to be derived has to fulfil two demands. First, the product of FDG activity and scan time per bed position should depend quadratically on a patient’s body weight. Second, specifications of RCs as described by EARL should be satisfied [8].

**Fig. 1:**
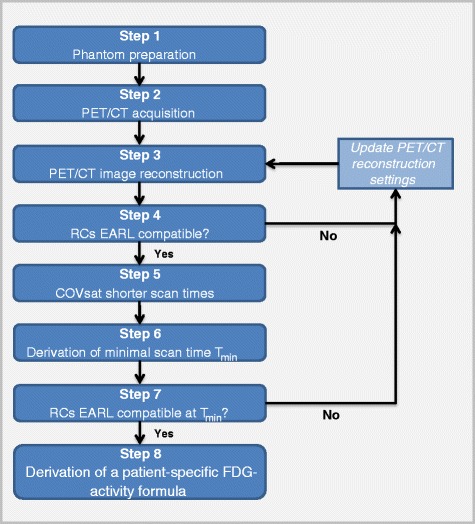
Flowchart demonstrating the eight steps to obtain a patient-specific FDG activity formula

In eight steps, we describe the method to derive this formula. Figure 1 shows a flow chart presenting all steps. A FDG-PET/CT phantom study using a NEMA NU2-2001 image quality phantom (IQ phantom) [9] forms the basis of our method.

### Step 1: phantom preparation

Prior to the phantom scan, the following materials should be available:

- A NEMA NU2-2001 IQ Phantom
- A bottle filled with 1000 mL water
- Two syringes, both with 20 MBq FDG activity (volume between 2 and 5 mL), specified at the expected phantom acquisition time *T*_a_ (hh:mm:ss).
- A dose calibrator

#### Filling of the spheres

- Measure the amount of FDG activity (in kBq) present in one syringe using the dose calibrator. Record the time of measurement *T*_m,1_ (hh:mm:ss) and record the volume of FDG activity present in the syringe (in mL).
- Add the FDG activity from this syringe to the bottle with water. Make sure all activity is entered into the bottle.
- Homogenize the solution in the bottle by shaking the phantom. Fill all phantom spheres with this solution [1].
- Calculate the true FDG activity concentration at the time of measurement in the spheres, [*S*_true_] at *T*_m,1_ (in kBq/mL), by dividing the FDG activity from the syringe at *T*_m,1_ as measured with the dose calibrator to the volume of the total solution of bottle and syringe [1].

Output

$$ \left[{S}_{\mathrm{true}}\right]\ \mathrm{at}\ {T}_{\mathrm{m},1}\kern0.5em \left(\mathrm{in}\ \mathrm{kBq}/\mathrm{mL}\right). $$

#### Filling of the background compartment

- Fill the background compartment of the IQ phantom completely with water.
- Remove 30 mL water from the background compartment.
- Measure the amount of FDG activity (in kBq) in the second syringe using the dose calibrator and record the time of measurement *T*_m,2_ (hh:mm:ss).
- Add the FDG activity from this syringe to the phantom background compartment. Make sure all activity is entered into the phantom. Homogenize the solution by shaking the phantom.
- Calculate the true FDG activity concentration in the phantom background compartment, [*B*_true_] at *T*_m,2_ (in kBq/mL), by dividing the FDG activity (in kBq) of the second syringe at *T*_m,2_ to the volume of the phantom background compartment [1].

Output

$$ \left[{B}_{\mathrm{true}}\right]\ \mathrm{at}\ {T}_{\mathrm{m},2}\kern0.5em \left(\mathrm{in}\kern0.5em \mathrm{kBq}/\mathrm{mL}\right). $$

### Step 2: PET/CT acquisition

- Position the IQ phantom on the scanner bed such that the centre of each sphere is located in a single transverse plane and at the centre of the axial field of view.
- Acquire a routine list-mode PET scan based on one bed position for at least 10 min, using a whole-body FDG-PET/CT protocol. Include a CT scan for attenuation correction purposes.
- Record the start time of the PET acquisition *T*_a_ and calculate the FDG activity concentrations in the spheres [*S*_true_] and the background compartment [*B*_true_] at *T*_a_. This can be done by correcting for FDG activity decay during the time between the FDG activity measurements *T*_m,1_ and *T*_m,2_ (step 1), and *T*_a_ [1].

Output

$$ \left[{S}_{\mathrm{true}}\right]\ \mathrm{at}\ {T}_{\mathrm{a}}\kern0.5em \left(\mathrm{in}\ \mathrm{kBq}/\mathrm{mL}\right)\ \mathrm{and}\kern0.5em \left[{B}_{\mathrm{true}}\right]\ \mathrm{at}\ {T}_{\mathrm{a}}\kern0.5em \left(\mathrm{in}\ \mathrm{kBq}/\mathrm{mL}\right). $$

### Step 3: PET/CT image reconstruction

Apply an image reconstruction that corrects for geometrical response and detector efficiency (normalization), system dead time, random coincidences, scatter and attenuation. In version 1.0 of the EANM procedure guidelines for tumour PET imaging, a number of indicative reconstruction settings are given for different system types [1].

### Step 4: EARL compatibility check

Based on the reconstructed image, measure the maximum and mean recovery coefficients (RCs) of the spheres, using the following definitions:

- The maximum activity concentration recovery coefficient (RC_max_) of a sphere is defined as the maximum pixel value within a sphere as measured on the reconstructed PET image, divided by the true FDG activity in the sphere [*S*_true_] at *T*_a_ [1].
- The mean activity concentration recovery coefficient (RC_mean_) of a sphere is determined by creating a volume of interest (VOI) at 50 % of the maximum pixel value, corrected for background uptake [1]. To obtain RC_mean_, the mean pixel value within this VOI is divided by the true FDG activity in the sphere [*S*_true_] at *T*_a_.

Check whether the measured RCs for all spheres are within the minimal and maximal RCs as defined by EARL [8]. If this is the case, continue to step 5. If not, go back to step 3 and revise the reconstruction settings, within the recommendations indicated in the EANM FDG-PET/CT procedure guidelines version 2 [2]. In general, by including or adapting a post-processing smoothing filter in the reconstruction, RCs can be reduced (by more filtering) or amplified (by less filtering) in such a way that they satisfy EARL requirements.

### Step 5: image coefficient of variation measurements at shorter scan times

- Perform additional reconstructions, using list-mode data and identical settings as determined at step 3, for shorter scan times at 75, 50, 25, 12.5, 6.25 and 3.13 % of the original scan duration of 10 min. Each reconstruction should be based on data with start time *T*_a_. In case re-reconstruction of data using list-mode acquisition is not possible, an alternative is to acquire multiple acquisitions, for example as described in the EARL procedure [10]. In that case, the scan time for each additional acquisition needs to be corrected for radioactive decay between the start time of the first acquisition *T*_a_ and the time of each next acquisition *T*_x_, using correction factor $C = {2}^{\left({T}_{\mathrm{x}}-{T}_{\mathrm{a}}\right)/{T}_{1/2}}$ with *T*_1/2_ is the half-life of fluor-18 (110 min).
- Create three rectangular regions of interest (ROIs), each of 900 mm^2^, in three axial planes within the phantom background compartment of the reconstructed images. For each ROI, the image coefficient of variation (COV) was determined by dividing the standard deviation to the mean pixel value within this ROI.
- The COV for a reconstructed image is obtained by taking the average of the nine measured COVs.

Output: COVs for images based on different scan times.

### Step 6: derivation of the minimal scan time *T*_min_

Create a graph comparing the COV on the *y*-axis with the scan time per bed position *T* (in seconds) on the *x*-axis. Include a power-law fit: COV = *a T*^−b^, with *a* and *b* as fit parameters. The minimal scan time per bed position (*T*_min_) can be derived using formula 1:

1 $$ {T}_{\min } = {\left(\frac{a}{{\mathrm{COV}}_{\max }}\right)}^{\frac{1}{b}}\cdot \frac{\left[{B}_{\mathrm{true}}\right]}{2.0} $$

In this formula, [*B*_true_] at *T*_a_ (in kBq/mL) is the true FDG activity concentration in the background compartment of the phantom at the start of the PET scan, as determined in step 2. In case [*B*_true_] deviates from 2.0 kBq/mL, the ratio [*B*_true_]/2.0 in formula 1 is necessary as in the EARL procedure [10], it is assumed that the background of the IQ phantom is filled with 2.0 kBq/mL FDG activity. An activity concentration of 2.0 kBq/mL would represent a patient with a reference body weight (*w*_ref_) of 75 kg, who received a reference FDG activity (*A*_ref_) of 300 MBq, 60 min prior to the scan time *T*_a_ [10]. Furthermore, a maximum COV (COV_max_) of 0.15 is proposed as a cut-off to set the minimal scan time [10].

Output

$$ {T}_{\min}\mathrm{a}\mathrm{t}\ \mathrm{a}\ \mathrm{predefined}\ {\mathrm{COV}}_{\max }. $$

### Step 7: EARL compatibility check at *T*_min_

Check whether the RCs are still within EARL specifications at *T*_min_.

- If this is the case, continue to step 8.
- If this is not the case, go back to step 3 and update the PET reconstruction settings, within the recommendations indicated in the EANM FDG-PET/CT procedure guidelines version 2.0 [2].

### Step 8: derivation of a patient-specific FDG activity formula

To determine the final FDG activity formula, the following input parameters are required:

- *T*_min_ (in seconds): the minimal scan time to reach COV_max_, as derived in step 6.
- *A*_ref_ and *w*_ref_: a reference FDG activity and reference body weight.

Formula 2 shows the formula for the product of FDG activity (*A* in MBq) to administer and the scan time *t* (in seconds) per bed position as applied in a clinical setting.

2 $$ A\cdot t=\frac{w^2}{w_{\mathrm{ref}}^2}\cdot {A}_{\mathrm{ref}}\cdot {T}_{\min } $$

The product *A* ⋅ *t* depends quadratically on a patient’s body weight and satisfies the EANM guideline in terms of RCs and COV.

Using *w*_ref_ = 75 kg and *A*_ref_ = 300 MBq as suggested by [10], formula 2 simplifies to:

3 $$ A\cdot t = 0.0533 \cdot {w}^2\cdot {T}_{\min } $$

## Results

We have tested the methodology described above using a state-of-the-art PET/CT scanner (Ingenuity TF, Philips Healthcare).

### Step 1: phantom preparation

We filled the IQ phantom with FDG activity. At *T*_m,1_ = 16:24:00, the concentration in the phantom spheres [*S*_true_] was 30.2 kBq/mL. Furthermore, the phantom background concentration [*B*_true_] was 2.4 kBq/mL at *T*_m,2_ = 16:58:00.

### Step 2: PET/CT acquisition

We performed a PET/CT scan which started at *T*_a_ = 17:38:00. Consequently, [*S*_true_] and [*B*_true_] at *T*_a_ were 18.9 and 1.84 kBq/mL, respectively.

### Step 3: PET/CT image reconstruction

**Fig. 2:**
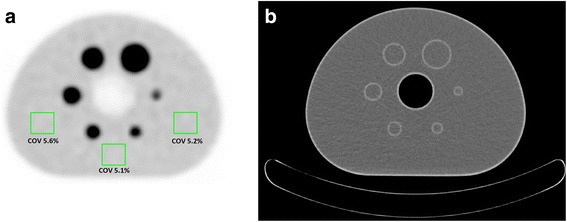
Phantom PET/CT images. Axial PET (**a**) and attenuation CT (**b**) images from the IQ phantom on the scanner bed. The phantom spheres and background were filled with FDG activity (ratio 10:1), and the scan duration was 10 min. The *squares* illustrate three ROIs in one axial plane that are used to determine the COV

We made a PET reconstruction using a default 3D ordered-subset iterative TOF reconstruction technique with 144 × 144 matrices (voxel size 4 × 4 × 4 mm^3^), 3 iterations, 43 subsets and a relaxation parameter 1.0 (“normal” smoothing setting), consistent with the reconstruction setting suggestions in the EANM guideline [1]. The reconstruction method is based on blobs, to compensate for detector blurring. The blob had a 2.5 mm radius, with a blob shape parameter of 8.4 mm. Figure 2 shows an axial PET and CT image of the IQ phantom filled with FDG.

### Step 4: EARL compatibility check

Mean and maximum RCs at 10 min scan duration are shown in Table 1. All RCs were within the EARL specifications.

**Table 1:** RC_mean_ and RC_max_ ranges as defined by EARL [8], compared with RC results for all spheres at 10 min and 62 s scan duration

Sphere volume (mL)	EARL: RC_mean_ range	RC_mean_ at *t* = 600 s	RC_mean_ at *t* = 62 s	EARL: RC_max_ range	RC_max_ at *t* = 600 s	RC_max_ at *t* = 62 s
26.52	0.76–0.89	0.79	0.80	0.95–1.16	0.98	1.05
11.49	0.72–0.85	0.75	0.74	0.91–1.13	0.96	1.04
5.57	0.63–0.78	0.72	0.69	0.83–1.09	0.97	0.94
2.57	0.57–0.73	0.68	0.64	0.73–1.01	0.93	0.90
1.15	0.44–0.60	0.44	0.48	0.59–0.85	0.59	0.71
0.52	0.27–0.38	0.33	0.27	0.31–0.49	0.44	0.40

For all spheres, RCs were within EARL specifications

### Step 5: image coefficient of variation measurements at shorter scan times

We used list-mode data with start time *T*_a_ to perform additional reconstructions with shorter scan durations and determined the COV from nine ROIs with three rectangular ROIs, as illustrated in Fig. 2, in three planes each.

### Step 6: derivation of the minimal scan time *T*_min_

**Fig. 3:**
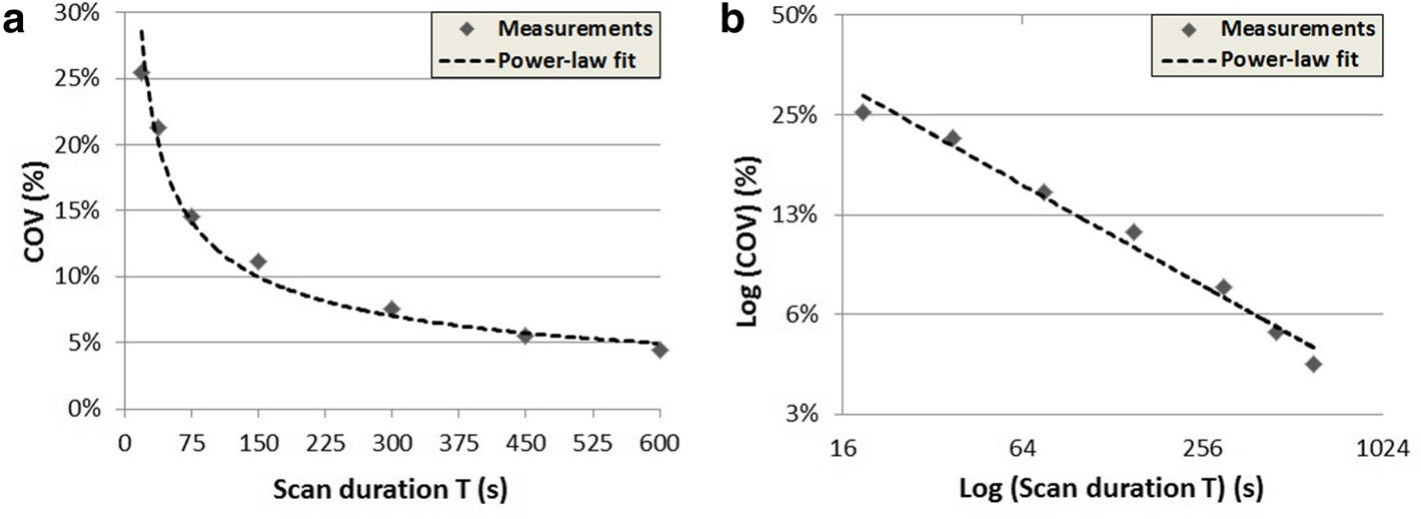
Comparing COV in the phantom background compartment measured at several scan durations, in graphs with standard scale (**a**) and log-log scale (**b**). A power-law fit resulted in COV = 1.26 *T* ^−0.51^. The coefficient of determination *r* ^2^ was 0.98, which indicates a good fit of the *trend line* to the data. Using the fit result, [*B*_true_] = 1.84 kBq/mL and COV_max_ = 0.15, formula 1 resulted in *T*_min_ = 62 s. The log-log scale graph can be described by log (COV) = log (*a*) − *b* · log (*T*) in which the steepness of the curve is described by (**b**)

In Fig. 3, the measured COVs are presented as a function of the scan duration. The values of the power-law fit parameters were *a* = 1.26 and *b* = 0.51. Using formula 1 with COV_max_ = 0.15 and [*B*_true_] = 1.84 kBq/mL, the minimal scan time *T*_min_ was found to be 62 s.

### Step 7: EARL compatibility check at *T*_min_

Mean and maximum RCs at *T*_min_ = 62 s scan duration are shown in Table 1. All RCs were within the EARL specifications.

### Step 8: derivation of a patient-specific FDG activity formula

Using formula 3 with *T*_min_ = 62 s, we derived the following body-weight-dependent formula for the product of FDG activity to administer and scan time per bed position.

4 $$ A\cdot t=\kern0.37em 3.29\ {w}^2 $$

## Discussion

The FDG activity formula presented in this paper provides a constant and standardized PET image quality for all patients [7]. Changing the value of COV_max_ will impact image quality and quantification accuracy. Ideally, its value should be chosen in such way that it provides the highest diagnostic accuracy. Note, however, that according to [10], COV_max_ should remain below 15 %, to keep image quality and quantification accuracy within acceptable limits. A lower COV_max_ value can easily be implemented in formula 1 and will result in higher FDG activity per patient, compared to the result based on a COV_max_ of 0.15. Furthermore, we used the EARL prescription that a phantom background compartment filled with 2.0 kBq/mL FDG activity represents a patient of 75 kg who received 300 MBq FDG activity. However, these reference values can be easily modified using formulas 1 and 2.

Our method includes a RC verification step on PET data acquired with the minimal scan time *T*_min_. This is important because it has been shown that an upward bias of (maximal) RCs can be expected at low scan statistics [11, 12]. In case RCs are above EARL requirements, it may therefore be helpful to apply an additional post-smoothing filter in the reconstruction that may compensate for this bias. It may also occur that an individual RC measurement does not fit EARL RC specifications due to statistical uncertainties at a shorter scan duration. When the difference between RCs and EARL requirements is relatively small, possibly after updating the reconstruction settings, it may also be useful to just repeat the reconstruction at a different time frame, e.g. starting at *T*_min_ and ending at 2 · *T*_min_.

Our suggested FDG activity formula provides an image quality that is achievable with multiple scanners at multiple PET centres. However, the reconstruction settings within this protocol are not necessarily optimized for optimal image quality. The latest generation PET scanners can provide an improved image quality. For example, the use of smaller voxels or point-spread function modelling, may improve the detection of small lesions [13, 14]. However, such reconstructions may also increase the image coefficient of variation and could therefore require a higher dose.

Furthermore, as already mentioned by de Groot et al. [7], the quadratic FDG activity regime results in very high levels of administered FDG activity for very heavy patients, when the scan time is not adapted. This may increase count rate losses of the system, and it increases the radiation burden for both the patient and the technician. Typically, it is recommended not to administer more than 530 MBq FDG activity for lutetium oxyorthosilicate systems [1, 6]. Using formula 4, with, e.g. an intrinsic scan time *t* of 90 s, this would imply that for patients with a body weight above 120 kg, it is advised not to further increase the administered FDG activity.

We derived the minimal scan time *T*_min_ by applying a power-law fit, to reduce the impact of single COV measurements at fixed time points. It can be discussed whether a power law is the best fit to describe the COV as a function of scan time. We assume that a power law can fit the data as noise properties in PET generally can be represented by a Poisson model, i.e. COV is generally inversely proportional to the square root of the measured counts. However, COV measurements, that are based on reconstructed data, may be influenced by detector dead time, normalization, attenuation correction or the reconstruction algorithm that is applied [4, 15]. Thereby, measured noise may not necessarily be represented by a Poisson model and hence a power-law fit may not be the best function to fit our COV data as a function of scan time. Furthermore, other techniques might be applied to estimate *T*_min_, for example by connecting the data points and reading the graph at a given COV_max_. In our study, the coefficient of determination *r*^2^ of 0.98 indicates a good fit of the power-law trend line to our data.

## Conclusions

This technical note provides a practical method that results in a formula describing the FDG activity to administer to individual patients for whole-body PET examinations, taking into account both the EANM guidelines and a quadric relation between FDG activity and a patient’s body weight.

## Abbreviations

[*B*_true_]: True FDG activity concentration in the phantom background
[*S*_true_]: True FDG activity concentration in the phantom spheres
*A*_ref_: Reference FDG activity
COV: Coefficient of variation
COV_max_: Maximal coefficient of variation
CT: Computed tomography
EANM: European Association of Nuclear Medicine
EARL: EANM Research Ltd
FDG: Fluor-18 fluordeoxyglucose
IQ phantom: NEMA NU2-2001 image quality phantom
PET: Positron emission tomography
RC_max_: Maximal activity concentration recovery coefficient
RC_mean_: Mean activity concentration recovery coefficient
ROI: Region of interest
*T*_a_: Time of acquisition
*T*_m_: Time of measurement
*T*_min_: Minimal scan duration per bed position
VOI: Volume of interest
*w*_ref_: Reference body weight
